# Different levels of blood pressure, different benefit from dual antiplatelet therapy in minor stroke or TIA patients

**DOI:** 10.1038/s41598-017-04169-8

**Published:** 2017-06-20

**Authors:** Jie Xu, Yongli Tao, Hao Li, Hongqiu Gu, Xuewei Xie, Xia Meng, Yuming Xu, Yilong Wang, Yongjun Wang

**Affiliations:** 1 Department of Neurology, Beijing Tiantan Hospital, Capital Medical University, Beijing, China; 2 China National Clinical Research Center for Neurological Diseases, Beijing, China; 3 Department of Neurology, the First Affiliated Hospital of Zhengzhou University, Zhengzhou, Henan China

## Abstract

The study aimed to evaluate whether the benefits of dual antiplatelet therapy would be influenced by blood pressure (BP) levels, among acute minor stroke or transient ischemic attack (TIA). In CHANCE (Clopidogrel in High-Risk Patients with Acute Nondisabling cerebrovascular Events) trail, Patients were stratified by systolic BP (SBP) and diastolic BP (DBP) level measured on admission, respectively, using the supine position BP within 24 hours after symptoms onset. The primary efficacy outcome was stroke recurrence, bleeding was the safety outcome. Patients with SBP ≥ 140 mmHg, dual antiplatelet treatment could reduce the risk of stroke recurrence significantly (HR 0.654, 95% CI 0.529–0.793, p < 0.001) than mono antiplatelet therapy. And patients with DBP ≥ 90 mmHg, clopidogrel-aspirin significantly reduced the risk of recurrent stroke (HR 0.588, 95% CI 0.463–0.746, p < 0.001), compared with aspirin alone. However, in patients with SBP < 140 mmHg or DBP < 90 mmHg, no significant difference was observed between clopidogrel plus aspirin and aspirin alone. there was no difference in bleeding episodes by treatment assignment across categories of SBP or DBP. Patients with SBP ≥ 140 mmHg or DBP ≥ 90 mmHg after minor stroke or TIA got more benefits from dual antiplatelet therapy. Bleeding risk from dual antiplatelet treatment did not increase among patients with higher BP level on admission.

## Introduction

Minor ischemic stroke and transient ischemic attack (TIA) patients are at high risk of recurrent stroke from 12% to 20% during the first 3 months after the index stroke or TIA^1–3^. The Clopidogrel in High-risk patients with Acute Nondisabling Cerebrovascular Events (CHANCE) trial found that dual antiplatelet therapy (clopidogrel plus aspirin) within 24 hours after symptom onset could reduce the risk of subsequent stroke by 32.0%, as compared with aspirin alone^4^. Besides dual antiplatelet therapy, antihypertensive therapy is also an effective intervention for prevention of recurrent stroke in minor stroke patients^5, 6^. As we know, the Secondary Prevention of Small Subcortical Strokes (SPS3) trial support that in patients with recent lacunar stroke, the use of a systolic-blood-pressure (SBP) target of less than 130 mm Hg is likely to be beneficial^7^. However, no studies had focused on whether these 2 intervention strategies have interaction on stroke outcomes.

In this subgroup analysis of the Clopidogrel in High-Risk Patients with Acute Nondisabling cerebrovascular Events (CHANCE) trail, we aimed to investigate whether there was interaction between BP (SBP and DBP) level and antiplatelet therapy on stroke recurrence among patients with minor stroke and high-risk TIA.

## Results

### Baseline Characteristics

There were four patients missing the SBP value among the total patients, so 5166patients were enrolled in the final analysis. Among the SBP ≥ 140 mmHg and SBP < 140 mmHg group, the baseline characteristics of mono and dual antiplatelet therapy were well balanced (Table 1). The baseline characteristics of patients stratified by DBP were shown in Table 1S (Supplementary Table S1).

**Table 1:** Baseline characteristics of patients with different SBP level.

**Variables**	SBP ≥ 140 mmHg			SBP < 140 mmHg		
	Clopidogrel-aspirin n = 1898	Aspirin n = 1892	P Value	Clopidogrel-aspirin n = 683	Aspirin n = 693	P Value
Age (median)	63	62	0.152	61	61	0.373
Female sex-no. (%)	639 (33.7)	670 (35.4)	0.259	212 (31.0)	227 (32.8)	0.495
BMI(median)	24	25	0.143	24	24	0.988
Medical history-no. (%)						
TIA OR Ischemic stroke	420 (22.1)	415 (21.9)	0.885	175 (25.6)	164 (23.7)	0.400
Myocardial infarction	28 (1.5)	37 (2.0)	0.254	15 (2.2)	16 (2.3)	0.888
Hypertension	1357 (71.5)	1359 (71.8)	0.820	358 (52.4)	323 (46.6)	0.031
Diabetes mellitus	391 (20.6)	394 (20.8)	0.865	158 (23.1)	149 (21.5)	0.467
Hypercholesterolemia	204 (10.7)	192 (10.1)	0.545	86 (12.6)	91 (13.1)	0.764
Current or previous smoking — no. (%)	816 (43.0)	788 (41.6)	0.403	299 (43.8)	317 (45.7)	0.463
Current or previous drinking- no. (%)	614 (32.3)	585 (30.9)	0.344	189 (27.7)	212 (30.6)	0.233
Qualifying event — no. (%)			0.668			0.939
TIA	487 (25.7)	497 (26.3)		229 (33.5)	231 (33.3)	
Minor stroke	1411 (74.3)	1395 (73.7)		454 (66.5)	462 (66.7)	
Secondary prevention						
anti-hypertension	761 (40.3)	733 (38.9)	0.365	166 (24.6)	153 (22.3)	0.326
lowering-lipid	803 (42.6)	815 (43.3)	0.672	291 (43.1)	260 (38.0)	0.053

There were four patients missing the systolic blood pressure values.

Abbreviations: BMI = body mass index; DBP = diastolic blood pressure; SD = standard deviation, TIA = transient ischemic attack.

### Efficacy and Safety Outcomes

**Figure 1:**
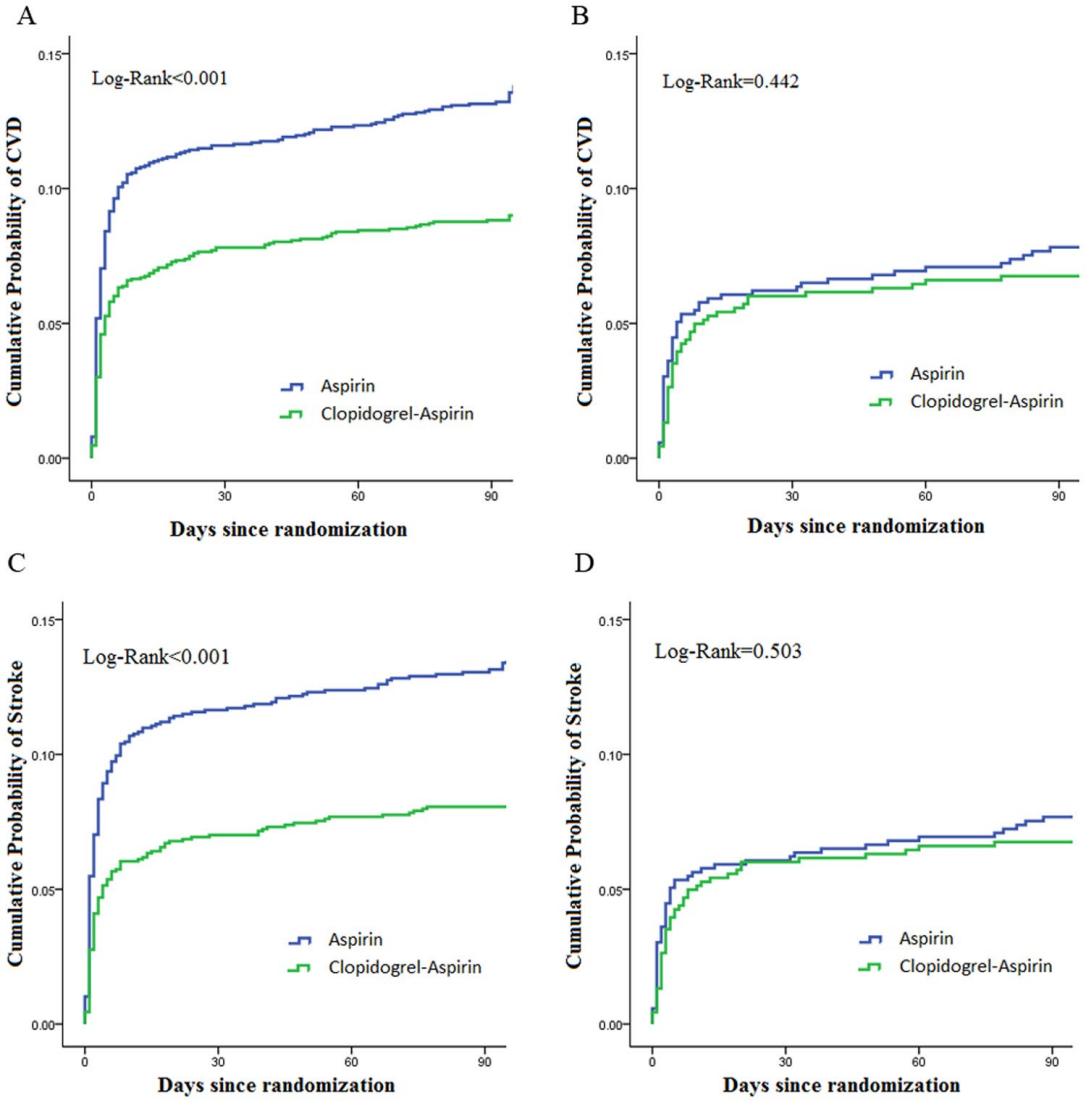
Kaplan-Meier curves showing the time to stroke outcomes in patients with SBP ≥ 140 mmHg or SBP < 140 mmHg, treated with clopidogrel-aspirin or aspirin alone. (**A**) Cumulative probability of CVD by treatment among patients with SBP ≥ 140 mmHg; (**B**) Cumulative probability of CVD by treatment among patients with SBP < 140 mmHg; (**C**) Cumulative probability of stroke by treatment among patients with SBP ≥ 140 mmHg; (**D**) Cumulative probability of stroke by treatment among patients with SBP < 140 mmHg. Abbreviations: CVD = combined vascular events (ischemic stroke, hemorrhagic stroke, myocardial infarction, or vascular death); SBP = systolic blood pressure.

**Figure 2:**
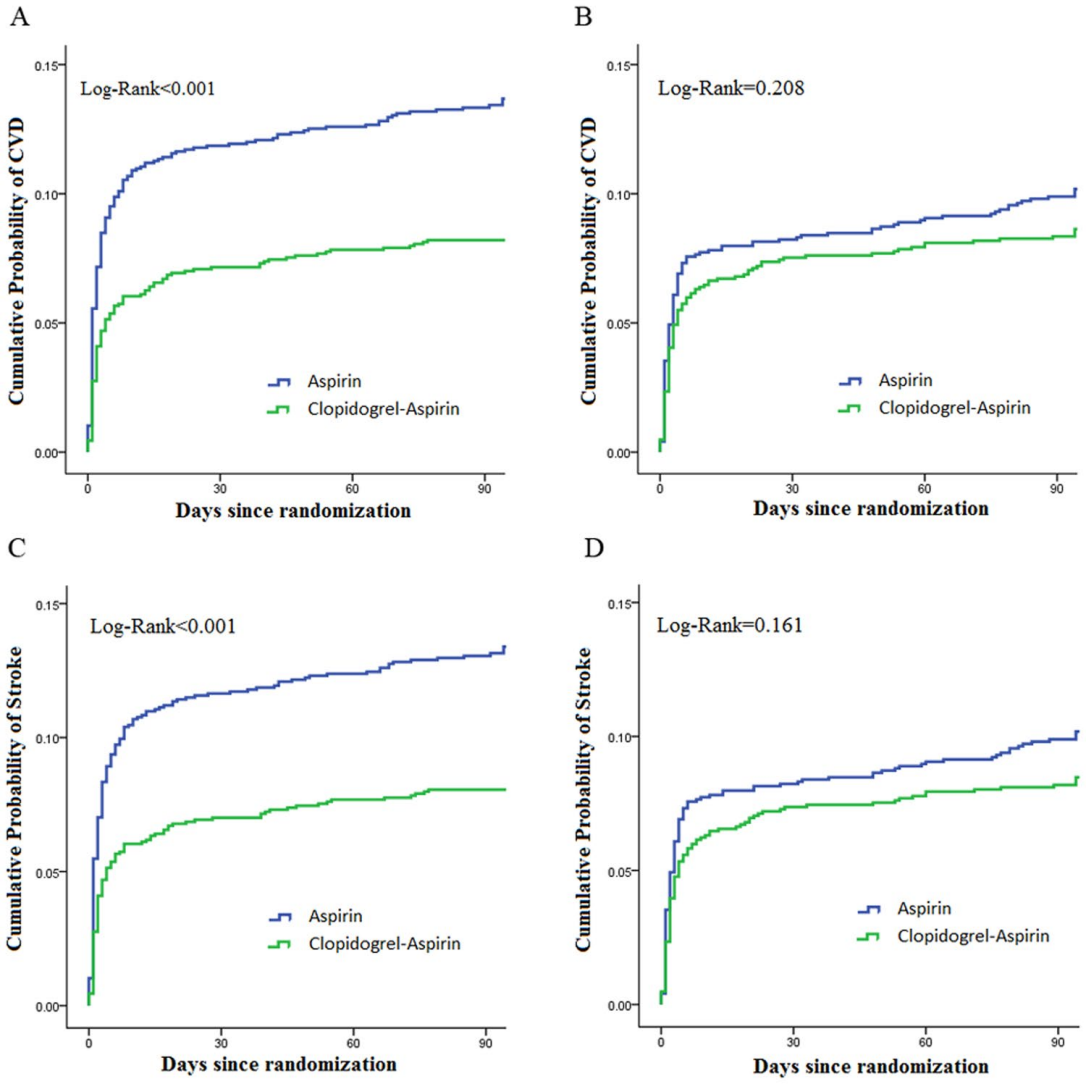
Kaplan-Meier curves showing the time to stroke outcomes in patients with DBP ≥ 90 mmHg or DBP < 90 mmHg, treated with clopidogrel-aspirin or aspirin alone. (**A**) Cumulative probability of CVD by treatment among patients with DBP ≥ 90 mmHg; (**B**) Cumulative probability of CVD by treatment among patients with DBP < 90 mmHg; (**C**) Cumulative probability of stroke by treatment among patients with DBP ≥ 90 mmHg; (**D**) Cumulative probability of stroke by treatment among patients with DBP < 90 mmHg. Abbreviations: CVD = combined vascular events (ischemic stroke, hemorrhagic stroke, myocardial infarction, or vascular death); DBP = diastolic blood pressure.

In patients with SBP < 140 mmHg, clopidogrel-aspirin could reduce the recurrent stroke (8.7% versus 13.2%, crude hazard ratio [HR] 0.654, 95% confidence interval [CI], 0.529–0.793, p < 0.001) and combined vascular events significantly (8.9% versus 13.4%, HR 0.651, 95% CI, 0.536–0.791, p < 0.001), compared with aspirin alone(Table 2 and Fig. 1). In addition, among patients with DBP ≥ 90 mmHg, dual antiplatelet therapy also significantly reduced the occurrence of new stroke (8.0 versus 13.3%, crude HR 0.588, 95% CI, 0.463–0.746, p < 0.001), and combined vascular events (8.2 versus 13.6%, crude HR 0.585, 95% CI, 0.462–0.741, p < 0.001) compared with mono antiplatelet treatment (Table 3 and Fig. 2).

**Table 2:** Effects of dual and mono antiplatelet therapy on outcomes by SBP levels.

Outcome	SBP mmHg	Clopidogrel-Aspirin Event rate (%)	Aspirin Event rate (%)	Crude		Adjusted	
				Hazard ratio (95% CI)	P value	Hazard ratio (95% CI)	P value*
Stroke	≥140	165 (8.7)	250 (13.2)	0.654 (0.529–0.793)	<0.001	0.639 (0.525–0.778)	<0.001
	<140	46 (6.7)	53 (7.6)	0.844 (0.559–1.258)	0.503	0.901 (0.517–1.339)	0.606
CVD	≥140	169 (8.9)	253 (13.4)	0.651 (0.536–0.791)	<0.001	0.647 (0.532–0.786)	<0.010
	<140	46 (6.7)	54 (7.8)	0.858 (0.579–1.271)	0.442	0.86 (0.596–1.311)	0.540
Bleeding	≥140	43 (2.3)	30 (1.6)	1.361 (0.852–2.175)	0.197	1.354 (0.847–2.165)	0.205
	<140	17 (2.5)	11 (1.6)	1.561 (0.731–3.332)	0.250	1.601 (0.746–3.434)	0.227

*Adjusted for gender, age, body mass index, history of TIA or ischemic stroke, hypertension, hypercholesterolemia, diabetes mellitus, current or previous smoking, moderate to heavy drinking, qualifying event, antihypertensive drugs, lipid-lowering agent and antidiabetic agent.

Abbreviations: CVD = combined vascular events (ischemic stroke, hemorrhagic stroke, myocardial infarction, or vascular death); SBP = systolic blood pressure.

**Table 3:** Effects of dual and mono antiplatelet therapy on outcomes by DBP levels.

Outcome	DBP mmHg	Clopidogrel-Aspirin Event rate (%)	Aspirin Event rate (%)	Crude		Adjusted	
				Hazard ratio (95% CI)	P value	Hazard ratio(95% CI)	P value*
Stroke	≥90	108 (8.0)	182 (13.3)	0.588 (0.463–0.746)	<0.001	0.597 (0.471–0.758)	<0.01
	<90	103 (8.3)	121 (10.0)	0.830 (0.638–1.080)	0.161	0.819 (0.629–1.065)	0.136
CVD	≥90	110 (8.2)	186 (13.6)	0.585 (0.462–0.741)	<0.001	0.595 (0.470–0.753)	<0.001
	<90	105 (8.5)	121 (10.0)	0.846 (0.652–1.099)	0.208	0.86 (0.643–1.085)	0.178
Bleeding	≥90	29 (2.2)	20 (1.5)	1.434 (0.811–2.535)	0.215	1.408 (0.796–2.489)	0.239
	<90	31 (2.5)	21 (1.7)	1.398 (0.801–2.443)	0.239	1.426 (0.816–2.494)	0.213

*Adjusted for gender, age, body mass index, history of TIA or ischemic stroke, hypertension, hypercholesterolemia, diabetes mellitus, current or previous smoking, moderate to heavy drinking, qualifying event, antihypertensive drugs, lipid-lowering agent and antidiabetic agent.

Abbreviations: CVD = combined vascular events (ischemic stroke, hemorrhagic stroke, myocardial infarction, or vascular death); DBP = diastolic blood pressure.

However, in patients with SBP < 140 mmHg or DBP < 90 mmHg, no significant difference was observed in the occurrence rate of recurrent stroke, and combined vascular events between clopidogrel -aspirin and aspirin alone (Figs 1 and 2).

After adjusting for gender, age, BMI, current or previous smoking and drinking, medical history (TIA or ischemic stroke, hypertension, hypercholesterolemia, diabetes mellitus), qualifying event (TIA or minor stroke), and secondary medication (anti-hypertension, anti-diabetes and lowering-lipid), the multivariable Cox proportional hazards models showed the similar results (Tables 2 and 3).

In addition, there was no significant difference of bleeding event rate between dual and mono antiplatelet treatment, either in SBP or DBP subgroup (Table 2 and 3).

## Discussion

In this post-hoc analysis of the CHANCE trial, we found that patients with SBP ≥ 140 mmHg or DBP ≥ 90 mmHg got more benefits from dual antiplatelet therapy on stroke recurrence and CVD than patients with SBP < 140 mmHg or DBP < 90 mmHg. And clopidogrel plus aspirin did not increase the risk of bleeding in patients with SBP ≥ 140 mmHg or DBP ≥ 90 mmHg.

Why the BP level could affect the efficacy of dual antiplatelet therapy on patients with minor stroke or TIA? The precise underlying mechanism remains unclear. Because baseline BP was measured within 24 hours after stroke onset, we considered that the protection mechanism^8–10^ of stress-induced hypertension after stroke may amplify the efficacy of dual antiplatelet therapy, especially for those with large artery stenosis in cerebral or carotid arteries. Previous studies^11, 12^ indicated that dual antiplatelet therapy with clopidogrel and aspirin was more effective than aspirin alone in reducing microembolic signals in patients with ischemic stroke or TIA due to extracranial or intracranial arterial stenosis. And patients with multiple stenosis of cerebral arteries need higher BP level to maintain the cerebral perfusion^13–15^. The synergistic effect between high BP and dual antiplatelet may contribute to the current finding.

Elevated BP upon admission was common in patients with acute ischemic stroke, which was considered to play an important role in maintain the cerebral circulation and cerebral perfusion of ischemic tissue^16, 17^. However, several previous studies also demonstrated that increased BP in acute phase may be detrimental to the brain edema and hemorrhage transformation^18–20^. So the relationship between early elevation in BP and prognosis remains controversial, some reports suggested that initially high blood pressure was associated with a poor prognosis^21–23^, while other study suggested that elevated BP contributed to a more favorable prognosis^8^ or did not affect the prognosis^24^. The previous study demonstrated that elevated BP was significantly associated with a past history of hypertension, which was consisted with our study (See Table 1)^17^. As we all known, hypertension is one of the most important risk factor for atherosclerosis and stroke^25^, so patients with past history of hypertension maybe combine more serious atherosclerosis than normotensive patients, and need higher BP level to maintain the cerebral perfusion of ischemic tissue. We suspect that synergies between the hypertension and dual antiplatelet may improve the prognosis.

In the present study, clopidogrel plus aspirin did not seem to increase the greater bleeding risk in patients with elevated BP on admission than patients with SBP < 140 mmHg or DBP < 90 mmHg. Although the exact mechanism underlying the conflicting results was not clear, and the possible explanation for the difference as follow. First, compared with previous studies including patients with more severe stroke, the patients in our trail at a relative low risk for bleeding or hemorrhage transformation. Second, in our trail, dual antiplatelet treatment was administered for just 21days, followed by clopidogrel alone for a total of 90 days. Dual antiplatelet therapy in short term did not increase the risk of bleeding.

Our study has some limitations. Firstly, not all patients had imaging data in CHANCE trail^26^, we could not get the information about extracranial or intracranial arterial stenosis of all the patients, so the above assumption cannot be validated in the study. Further investigation is needed to investigate the synergistic effect between BP and dual antiplatelet therapy in ischemic stroke patients with symptomatic extracranial or intracranial arterial stenosis. Secondly, the incidence of bleeding events was lower in CHANCE trail, which may reduce the statistical power. So the future study need to further manifested the relationship between very high BP after ischemic stroke and bleeding risk, among patients who received dual antiplatelet treatment. Third, this study was a post-hoc analysis, which may increase the type I error, so our result need to be confirmed by other studies.

In conclusion, we found an interesting phenomenon, which may give some implications for clinical practice. That is, minor stroke or TIA patients with SBP ≥ 140 mmHg or DBP ≥ 90 mmHg, maybe more suitable for dual antiplatelet therapy. With respect to safety outcome, bleeding risk of dual antiplatelet therapy did not seem to increase with elevated BP.

## Methods

### Study population

CHANCE was a randomized, double-blind, placebo-controlled clinical trial at 114 clinical centers in China as reported elsewhere^4, 27^. In brief, patients ≥ 40 years with acute minor stroke (NIH Stroke Scale < [NIHSS]^28^ ≤3) or high-risk TIA (ABCD^2^ ≥4) within 24 hours of symptoms ictus were randomly assigned to either dual antiplatelet treatment (clopidogrel at an initial dose of 300 mg, followed by 75 mg per day for 90days, plus aspirin at a dose of 75 mg per day for the first 21 days) or mono antiplatelet treatment (aspirin at a dose of 75 mg per day for 90 days). The CHANCE protocol was approved by ethics committees of Beijing Tiantan Hospital and all other study centers. All participants or their legal proxies provided written informed consent.

### Blood Pressure Measurement and Group Assignment

At admission, within 24 hours after symptoms ictus, three BP readings separated by at least two minutes in the supine position were recorded by doctors or trained nurses. Measurement of BP was according to a protocol for BP measurement in the clinic or office recommended by the American Heart Association^29^, and the average of the three readings as the BP on admission.

In this subgroup analysis, we tested the efficacy and safety of dual antiplatelet therapy in patients stratified by the BP level on admission: SBP ( ≥140 mmHg or < 140 mmHg),and DBP ( ≥90 mmHg or < 90 mmHg), respectively.

### Outcomes assessment

The efficacy outcomes^4^ were stroke recurrence (ischemic stroke or hemorrhage stroke), and combined vascular events (ischemic stroke, hemorrhagic stroke, myocardial infarction, or vascular death) during 90 days of follow-up. The safety outcome was any bleeding, according to the Global Utilization of Streptokinase and Tissue Plasminogen Activator for Occluded Coronary Arteries (GUSTO) definition^30^. Moreover, the follow-up at 90-day was done by face to face. All of the efficacy and safety outcomes were confirmed by the central adjudication committee which was blinded to the study group assignments.

### Statistical analysis

We used SAS version 9.4 (SAS Institute Inc, Cary, NC) for all statistical analyses. For descriptive analysis, proportions and medians were used for categorical and continuous variables. The baseline characteristics among dual and mono antiplatelet therapy in BP subgroup were compared by Chi-square tests or Fisher exact test for categorical variables and continuous variables were compared by Kruskal-Wallis test. For each group stratified by BP level, the differences between dual and mono antiplatelet therapy in the rates of stroke recurrence, combined vascular events, and bleeding during the 90 days follow up were evaluated by using crude and multivariable Cox proportional hazards models. Two-tailed p value less than 0.05 indicated statistical significance.

## Electronic supplementary material

Table S1
