# Supplementary material for: Different levels of blood pressure, different benefit from dual antiplatelet therapy in minor stroke or TIA patients
Source: Sci Rep. 2017 Jun 20;7:3884. doi: 10.1038/s41598-017-04169-8 (PMC5478626; doi:10.1038/s41598-017-04169-8)
Supplement: Supplementary file 1 — Table S1 [file 41598_2017_4169_MOESM1_ESM.pdf]

TITLE PAGE

TITLE:

Different levels of blood pressure, different benefit from dual antiplatelet therapy in minor stroke or TIA patients

SHORT TITLE: blood pressure and benefits from dual antiplatelet treatment

AUTHOR LIST

Jie Xu*, Yongli Tao*, Hao Li, Hongqiu Gu , Xuewei Xie , Xia Meng, Yuming Xu , Yilong Wang, Yongjun Wang

***** These authors contributed equally to the manuscript

Table 1s. Baseline characteristics of patients with different DBP levels

Variables	DBP≥90			DBP＜90		
	Clopidogrel-aspirin n= 1343	Aspirin n= 1368	P Value	Clopidogrel-aspirin n=1238	Aspirin n=1216	P Value
Age (median)	60.14	59.94	0.617	64.98	64.56	0.070
Female sex-no.(%)	420 (31.3)	455 (33.3)	0.269	431 (34.8)	442(36.3)	0.427
BMI (median)	24.65	24.80	0.471	24.22	24.34	0.353
Medical history-no.(%)						
TIA OR Ischemic stroke	302 (22.5)	273 (20.0)	0.107	293 (23.7)	305 (25.1)	0.414
Myocardial infarction	16 (1.2)	22 (1.6)	0.355	27 (2.2)	31 (2.5)	0.548
Hypertension	978 (72.8)	974 (71.2)	0.347	737 (59.5)	708 (58.2)	0.510
Diabetes mellitus	241 (17.9)	250 (18.3)	0.824	308 (24.9)	293 (24.1)	0.652
Hypercholesterolemia	139 (10.3)	130 (9.5)	0.461	151 (12.2)	153 (12.6)	0.772
Current or previous smoking -no. (%)	612 (45.6)	600 (43.9)	0.371	503 (40.6)	504 (41.4)	0.681
Current or previous drinking - no. (%)	467 (34.8)	463 (33.8)	0.611	336 (27.1)	334 (27.5)	0.856
Qualifying event — no. (%)			0.883			0.490
TIA	342 (25.5)	345 (25.2)		374 (30.2)	383 (31.5)	
Minor stroke	1001 (74.5)	1023 (74.8)		864 (69.8)	833 (68.5)	
Secondary prevention						
anti-hypertension	548 (41.0)	536 (39.4)	0.387	379 (30.9)	350 (29.0)	0.296
lowering-lipid	580 (43.4)	583 (42.8)	0.762	514 (42.0)	492 (40.8)	0.549

There were five patients missing the DBP value.

Abbreviations: BMI=body mass index; DBP=diastolic blood pressure; SD=standard deviation, TIA=transient ischemic attack.
